# Effect of nitrate supplementation on diurnal emission of enteric methane and nitrous oxide

**DOI:** 10.3168/jdsc.2023-0541

**Published:** 2024-05-17

**Authors:** W. Wang, M. Larsen, M.R. Weisbjerg, A.L.F. Hellwing, P. Lund

**Affiliations:** Department of Animal and Veterinary Sciences, AU Viborg–Research Centre Foulum, Aarhus University, DK 8830 Tjele, Denmark

## Abstract

The objective of this study was to investigate the effect of nitrate supplementation on diurnal enteric methane (CH_4_) and nitrous oxide (N_2_O) emissions in dairy cows. Four Danish Holstein dairy cows fitted with ruminal cannulas were used in a 2 × 2 crossover design with 2 periods of 14 d duration. Cows were fed ad libitum with 2 experimental diets based on either urea or nitrate (8.6 g of ${NO}_{3}^{-}/kgofDM)$ supplementation. Samples of ruminal fluid, blood, and rumen headspace gas samples were collected. Gas exchange was measured in respiration chambers during a 96-h period. Emission of N_2_O was calculated from the ratio between CH_4_ and N_2_O in the rumen headspace and the measured CH_4_ emission. Nitrate supplementation resulted in a lower daily CH_4_ production (g/d), CH_4_ yield (g/kg of DMI), and CH_4_ per kilogram of fat- and protein-corrected milk yield; a tendency of lower CH_4_ intensity (g/kg ECM); and higher daily hydrogen (H_2_) production, H_2_ yield, and daily N_2_O production compared with urea supplementation. The only difference in ruminal VFA composition was a higher valerate proportion in cows receiving nitrate compared with urea supplementation. In conclusion, nitrate compared with urea supplementation reduced CH_4_ production, mainly just after feeding, but also increased N_2_O production.

## Highlights

• Nitrate supplementation reduced CH_4_ production and yield and increased H2 production and yield compared with urea supplementation.
• Nitrate supplementation increased N_2_O production compared with urea supplementation.
• The diurnal patterns of methane production (chambers) and methane volume proportion (rumen headspace) reflect each other.

## Graphical Abstract

**Figure:**
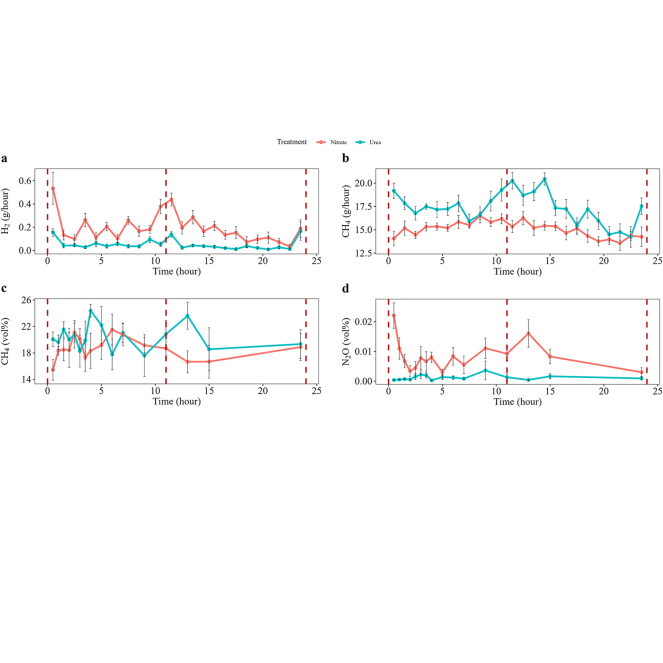
**Summary:** Nitrate has been extensively studied and is confirmed to have a significant methane mitigation effect in ruminants. However, it is proven that nitrate supplementation can stimulate the emission of nitrous oxide. In this study, we investigated the effect of nitrate supplementation on ruminal fermentation and related emissions of CH_4_, CO_2_, and H_2_ on cow level, CH_4_, CO_2_, and N_2_O on rumen level, and prediction of N_2_O on cow level from rumen volume proportion. The results suggested that nitrate supplementation reduced CH_4_ production and yield, with the largest effect on production just after feeding, and increased H_2_ production and yield compared with urea supplementation. However, nitrate supplementation also increased N_2_O production compared with urea supplementation.

Methane emission from ruminants results in notable losses of energy and is an important GHG that contributes to global warming. Previous studies have shown that nitrate has a promising CH_4_ mitigation effect in dairy cows (Olijhoek et al., 2016; Wang et al., 2023). Unfortunately, a higher N_2_O production has also been noted when nitrate was applied to reduce CH_4_ emission (Petersen et al., 2015). A better understanding of the effect of nitrate supplementation on N_2_O emission from the rumen is crucial to assess the net global warming mitigation effect of nitrate supplementation in dairy cows, because N_2_O might counteract the reduction in CO_2_ equivalents as N_2_O has a higher global warming potential than CH_4_. The N_2_O emission from cows is very low (Petersen et al., 2015), and instruments able to measure the low concentrations are expensive. An alternative approach would be to measure the headspace concentration of N_2_O and relate it to the CH_4_ emission measured in the chamber. To our best knowledge, no previous study has looked into the N_2_O emission based on rumen headspace samples. The respiration chamber is considered the gold standard for measuring CH_4_ emission from an individual cow (Huhtanen et al., 2015), while a more detailed investigation on the diurnal pattern of gas production in the rumen could provide valuable insight into the relationship between nitrate supplementation and CH_4_ production in the rumen. The objective of this study was to investigate the effect of nitrate supplementation on ruminal fermentation and related emissions of CH_4_, CO_2_, and H_2_ on cow level; CH_4_, CO_2_, and N_2_O on rumen level; and prediction of N_2_O on cow level from rumen concentration. Therefore, we hypothesized that replacing urea with nitrate would cause an increased N_2_O production in the rumen at the same time as it reduces CH_4_ production.

Four multiparous (2 each of second and third parities) Danish Holstein cows fitted with rumen cannulas (#4C, Bar Diamond Inc.) were used in a crossover design with two dietary treatments and two 14-d periods. Between chamber measurements, cows were loose housed individually in housing pens with cubicles and had free access to water. Cows were milked twice daily at 0515 and 1630 h. Milk was sampled, analyzed, ECM was calculated as described by Wang et al. (2023), and fat- and protein-corrected milk yield (**FPCM**) was calculated as described by Daniel et al. (2020). At the beginning of the experiment, BW, DIM, and milk yield averaged (mean ± SD) 676 ± 49 kg, 199 ± 73 d, and 29.4 ± 8.9 kg/d, respectively.

The 2 diets were fed as TMR and consisted of (DM basis) 33.4% corn silage, 22.4% grass-clover silage (spring growth), 14.1% beet pulp (dried), 13.7% rapeseed cake, 9.42% spring barley, 4.32% soybean meal, 0.79% sodium bicarbonate, 0.47% commercial mineral and vitamin mixtures, and 1.4% of isonitrogenous urea or nitrate supplement. The urea supplement was 48% urea-mix (80% urea, 13.5% sodium sulfate, 6.5% calcium carbonate; Vilofoss) and 52% calcium carbonate and the nitrate supplement was 95% SilvAir [5Ca(NO_3_)_2_·NH_4_NO_3_·10H_2_O; 750 g of ${NO}_{3}^{-}/kgofDM;$ Cargill Inc.] and 5% sodium sulfate. Chemical composition of the diets was 6.88% and 6.77% of ash, 16.0% and 16.1% of CP, 31.6% and 31.3% of NDF, and 3.50% and 3.45% of crude fat for urea and nitrate supplemented diets, respectively.

The diets were fed ad libitum twice daily (0540 and 1650 h), and residues were removed and weighed before the morning feeding. Cows were fed and milked in the same order with a 15-min interval between each cow during the chamber measurement period, to fit the sampling process. The TMR offered and orts were sampled.

A ruminal fluid sample was collected at 0830, 1130, and 1430 h on d 10 before cows entered the chambers. Ruminal fluid sample collection was described by Olijhoek et al. (2016). A blood sample was collected on d 10 at 0830 h by venipuncture of the coccygeal vein into sodium heparin Vacutainers.

Gas exchange was measured using 4 respiration chambers. The respiration chambers were larger compared with the description in Hellwing et al. (2012) and slurry pits and air locks were installed. Cows entered the chambers before morning feeding on d 11 and the gas exchange was measured for 96 h. Recoveries of CH_4_ measured before and after experiment were 100 ± 0.29%.

Sixteen spot samples of rumen headspace gas were collected on d 12 and on d 14 at 0600, 0630, 0700, 0730, 0800, 0830, 0900, 0930, 1030, 1130, 1230, 1430, 1630, 1830, 2030 h, and at 0530 h on d 13 and 15 to give frequent measures after morning feeding. The rumen headspace gas was sampled in a staggered way with a 15-min interval between each cow and following the same order as milking and feeding. Therefore, the actual time lapse from feeding is the same for all cows at a given sampling time. The rumen headspace gas samples were collected using a sampling device composed of a 30-cm steel rumen sampler (Bar Diamond Inc.) with a 50-mesh stainless steel screen at one end, and the other end was fitted with an injection site adapter (Medline Industries Inc.) for connecting the syringe. The gas sampler was bended in a “U” shape to ensure that the steel screen end is located dorsal in the headspace when inserted through the rumen cannula. A small piece of sponge was immediately tucked in the small hole of the rumen cannula once the sampler was properly placed in the rumen headspace. A 10-mL syringe was connected to the sampler, and the first 10 mL of extracted gas was discarded to reduce atmospheric air contamination. Two 10-mL samples of rumen headspace gas were collected and immediately injected separately into 2 pre-evacuated 5.9 mL vials (Labco Exetainer, Labco).

The DM was determined in TMR and orts using a forced-air oven at 60°C for 48 h. Contents of ash, N, crude fat, and NDF were analyzed as described by Wang et al. (2022).

Ruminal fluid was analyzed for pH, VFA, and NH_3_ as described by Wang et al. (2022). Redox potential in ruminal fluid was measured by inserting a redox probe (MTC101, HACH) into the ventral rumen. The hemoglobin (**Hb**) and methemoglobin (**MetHb**) content in blood were analyzed as described by Wang et al. (2023).

Concentrations of CH_4_, CO_2_, and H_2_ in the gas samples were analyzed as described by Thorsteinsson et al. (2023). Concentrations of N_2_O were analyzed by GC-ECD (Thermo Fisher Scientific TRACE 1310) equipped with a headspace auto-sampler (TriPlus RSH, Thermo Fisher Scientific). The system used a TG-BOND Q column (30 m × 0.32 mm × 10 µm; Thermo Fisher Scientific) and He as carrier gas. The injector temperature was 150°C and the initial oven temperature was held at 40°C for 1.75 min and increased to 150°C with a rate of 20.0°C/min and then held for 1 min. The pressure of the column was held at 0.12 MPa.

Crude protein was calculated as total N × 6.25. Data on DMI (from gas exchange period) and gas exchange were averaged per cow over the last 4 d within each period.

Volume proportions of CO_2_, CH_4_, and N_2_O in ruminal headspace gas samples were calculated and corrected for contamination with atmospheric air (N_2_, O_2_) assuming that CO_2_, CH_4_, and N_2_O made up 100% of true rumen headspace gases. Daily N_2_O production was calculated by using the following equation: daily N_2_O production (L/d) = daily CH_4_ production (L/d) × N_2_O:CH_4_ volume ratio (L/L), and recalculated to grams using the ideal gas law.

Data were analyzed in R (version 3.6.3) using the Fit Linear Mixed-Effects Models (LMM) through the ‘lmer' function in ‘lme4' package (Bates et al., 2015). The following model was used for DMI, gas emission, and milk yield and composition data:

*Y_ijk_* = *μ* + *TREATMENT_i_* + *PERIOD_j_* + *COW_k_* + *E_ijk_*,

where *Y_ijk_* is the dependent response variable; *μ* is the overall mean; *TREATMENT_i_* and *PERIOD_j_* are the fixed effects of treatment and period and *COW_k_* is the random effect of cows (*k =* 1 to 4); *E_ijk_* is the random residual error.

Data within cow and period were considered as repeated measurements and an autoregressive covariance structure (corAR1) was imposed via the R package ‘nlme' (Pinheiro et al., 2020). Hourly respiration chamber CH_4_ and H_2_ emission data, rumen headspace gas volume proportion data, and rumen fermentation parameters data were analyzed using the following model:

*Y_ijklm_ = μ + TREATMENT_i_* × *TIME_j_ + PERIOD_k_ + COW_l_ + DAY_m_ + E_ijklm_*,

where *TREATMENT_i_*, *TIME_j_*, and *PERIOD_k_* are the fixed effects of treatment, time spots, and period, respectively, and *COW_l_* and *DAY_m_* are the random effects of cows and sampling days in each period; *E_ijklm_* is the random residual error. Due to lack of normality of data, N_2_O volume proportion data in rumen headspace were log-transformed before statistical analysis and results presented in Table 1 were back transformed from the LSMEANS. Least squares means are presented, and significance or tendency was declared at *P* ≤ 0.05 and 0.05 < *P* ≤ 0.10, respectively.

**Table 1:** Gas exchange in respiration chamber and rumen headspace volume proportions in dairy cows with urea or nitrate supplementation (n = 4 for each treatment [Trt])

Item	Urea	Nitrate	SEM	*P* (Trt)	*P* (Time)	*P* (Trt × time)
DMI (kg/d)	22.9	23.0	1.68	0.95	—	—
Gas exchange in respiration chamber						
CH_4_ (g/d)	412	357	19.6	0.04	—	—
CH_4_ 1 (g/h)	17.4	15.1	0.80	<0.01	0.02	0.06
CH_4_/DMI (g/kg)	18.1	15.6	0.62	0.02	—	—
CH_4_/ECM (g/kg)	16.2	14.0	2.13	0.06	—	—
CH_4_/FPCM (g/kg)	17.7	11.2	0.59	<0.01	—	—
CH_4_/GEI (%)	5.31	4.56	0.18	0.02	—	—
H_2_ (g/d)	1.15	4.61	0.54	<0.01	—	—
H_2_ 1 (g/h)	0.06	0.19	0.02	<0.01	0.41	0.65
H_2_/DMI (g/kg)	0.05	0.21	0.03	<0.01	—	—
CO_2_ (g/d)	14,541	14,944	951	0.27	—	—
O_2_ (g/d)	9,423	9,625	604	0.33	—	—
Gas composition in rumen headspace						
CO_2_ (vol %)	79.5	81.3	1.89	<0.01	0.74	0.32
CH_4_ (vol %)	20.5	18.7	1.89	<0.01	0.74	0.32
N_2_O (vol %)	0.001	0.008	0.00	<0.01	0.70	0.48
N_2_O production^2^ (g/d)	0.10	0.47	0.03	<0.001	—	—

1 Hourly measurement from respiration chamber.

2 Calculated based on the CH_4_ production measured by respiration chamber and the N_2_O:CH_4_ volume proportion ratio in the rumen headspace.

The DMI, ECM yield, and milk composition did not differ between treatments (*P* > 0.05). The average ECM yield and percentage of fat, protein, and lactose were 27.2 ± 3.90 kg/d, 4.54% ± 0.43%, 3.91% ± 0.26%, and 4.71% ± 0.10%, respectively, for cows receiving urea supplementation, and 27.2 ± 3.90 kg/d, 4.51% ± 0.43%, 3.94% ± 0.26%, and 4.75% ± 0.10%, respectively, for cows receiving nitrate supplementation.

Daily CH_4_ production, yield, per kilogram of FPCM, and percentage of gross energy intake were 13.3%, 13.8%, 36.7%, and 14.1% lower for nitrate compared with urea supplementation (*P* = 0.04, *P* = 0.02, *P* < 0.01, and *P* = 0.02, respectively; Table 1). Nitrate supplementation tended to give lower CH_4_ intensity compared with urea supplementation (*P* = 0.06). Correspondingly, nitrate supplementation increased daily H_2_ production and H_2_ yield by 3.65 g/d and 0.16 g/kg DMI compared with urea supplementation (*P* ≤ 0.01). Aligned with the results of daily gas emission, cows receiving nitrate supplementation also showed a lower average hourly CH_4_ production but higher average hourly H_2_ production than cows receiving urea supplementation in the respiration chamber measurement (both *P* < 0.01). In addition, a tendency for an interaction between nitrate supplementation and time on the hourly CH_4_ production was noted (*P* = 0.06). Hourly CH_4_ production from cows receiving urea supplementation peaked shortly after feeding, whereas less fluctuation was observed in cows receiving nitrate supplementation. Nitrate supplementation resulted in a 5-fold higher N_2_O production (0.47 g/d) compared with urea supplementation (0.10 g/d; *P* < 0.001). The N_2_O production is still small, but the global warming potential of N_2_O compared with CH_4_ is 273 versus 27 (IPCC, 2021), thereby counteracting around 7% of the CH_4_ reduction obtained by nitrate addition.

Cows receiving nitrate supplementation showed a lower rumen headspace CH_4_ volume proportion (*P* < 0.01; Table 1 and Figure 1c) and greater rumen headspace CO_2_ and N_2_O volume proportions (*P* < 0.01) compared with cows receiving urea supplementation. Cows receiving nitrate supplementation showed a persistent lower hourly CH_4_ production and persistent higher hourly H_2_ production compared with cows received urea supplementation over the day (Table 1, Figure 1a).

**Figure 1:**
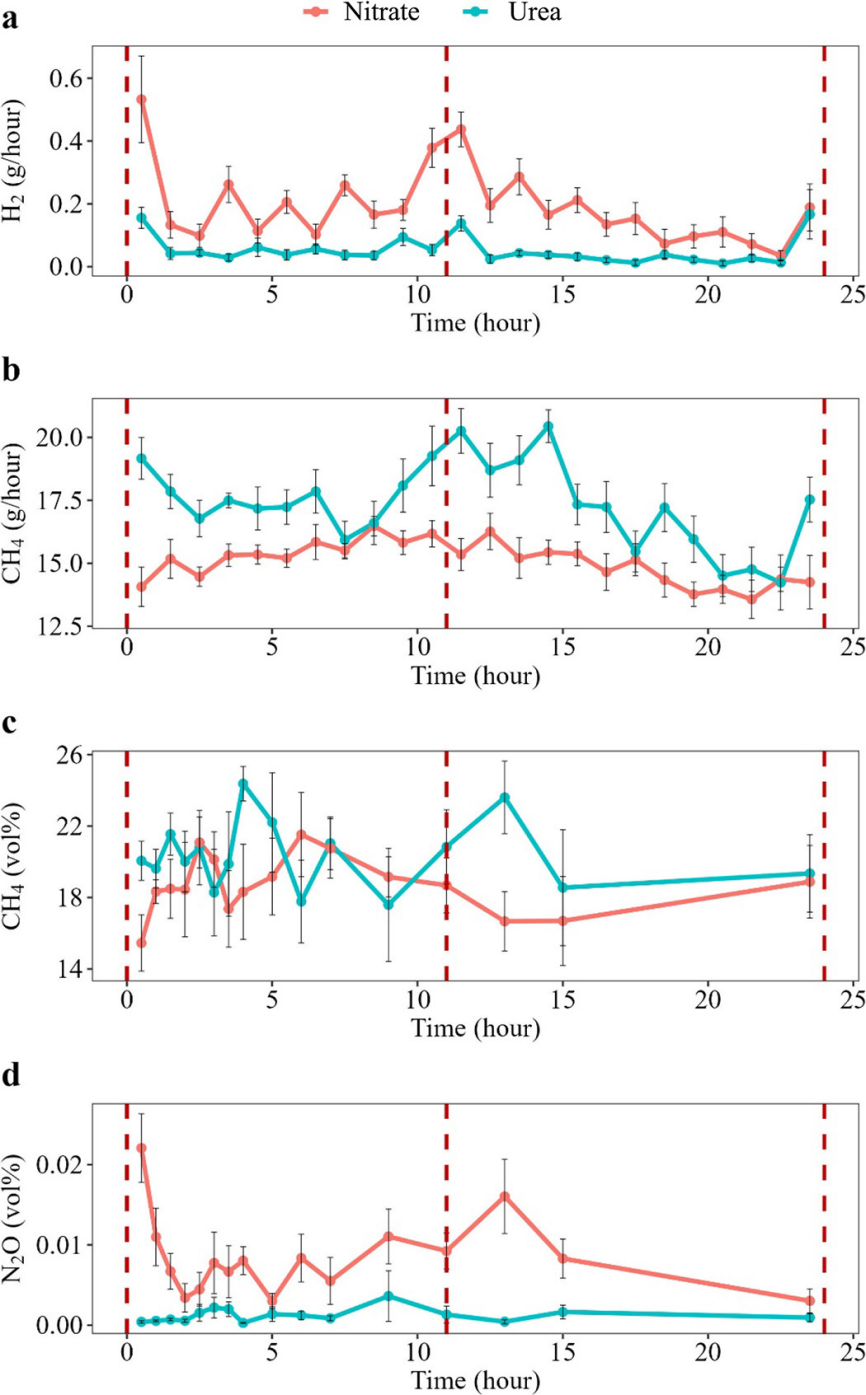
Diurnal pattern in (a) H_2_ and (b) CH_4_ emission in respiration chambers (g/h), and (c) CH_4_ and (d) N_2_O volume proportions (vol%) in rumen headspace. The x-axis is time related to morning feeding (dashed line indicates feeding). Values are LSM, and the error bars are SE of LSM. Statistics for the diurnal patterns are given in Table 1.

The ruminal total VFA concentration and molar proportion of individual VFA did not differ between urea or nitrate supplementation (*P* > 0.05; Table 2), except for a higher valerate proportion with nitrate supplementation (*P* = 0.02). Ruminal NH_3_ concentration, pH, and redox potential did not differ between treatments (*P* > 0.05). Concentration of Hb and MetHb proportion of total hemoglobin in blood did not differ between treatments (*P* > 0.05) and averaged 6.71 ± 0.36 mmol/L and 2.19 ± 0.06%, respectively, for urea supplementation and 6.80 ± 0.36 mmol/L and 2.10 ± 0.06%, respectively, for nitrate supplementation.

**Table 2:** Ruminal fermentation characteristics in dairy cows with urea or nitrate supplementation (n = 2 for each treatment [Trt])

Item	Urea	Nitrate	SEM	*P* (Trt)	*P* (Time)	*P* (Trt × time)
Total VFA (mmol/L)	125	123	3.58	0.67	0.03	0.85
VFA (mol/100 mol)						
Acetate (A)	59.9	59.6	1.04	0.79	0.64	0.79
Propionate (P)	21.4	20.6	0.93	0.20	0.77	0.72
Isobutyrate	0.70	0.67	0.05	0.23	0.02	0.35
Butyrate	13.9	14.7	0.54	0.10	0.40	0.26
Isovalerate	1.41	1.40	0.33	0.91	0.96	0.98
Valerate	1.88	2.11	0.22	0.02	0.44	0.27
Caproate	0.78	0.86	0.07	0.28	0.63	0.32
A:P ratio	2.82	2.93	0.16	0.36	0.82	0.88
NH_3_ (mmol/L)	5.44	4.50	1.06	0.54	0.68	0.80
Ruminal pH	6.28	6.38	0.07	0.29	0.14	0.93
Redox (mV)	−216	−221	12.9	0.71	0.68	0.29

Substantial mitigation effects of nitrate supplementation on CH_4_ production (13.3%) and CH_4_ yield (13.8%) were found in the current study as also reported in previous studies (Olijhoek et al., 2016; Wang et al., 2023). In theory, supplementation of 8.6 g of nitrate $\left({NO}_{3}^{-}\right)$ per kg of DM would result in a reduction of 2.22 g of CH_4_/kg of DMI if ${NO}_{3}^{-}$ is completely reduced to ${NH}_{4}^{+}.$ The 2.5 g/kg DMI reduction in CH_4_ yield was equivalent to 113% of the stoichiometric CH_4_ reduction potential of nitrate level (8.6 g of ${NO}_{3}^{-}/kgofDM)$ applied in the current study (Ungerfeld and Kohn, 2006; Maigaard et al., 2024). The CH_4_ mitigation efficacy of nitrate supplementation in the current study was higher than the values reported in most previous studies. Likewise, in our recent study, we observed an average CH_4_ yield reduction of 137% of theoretical stoichiometric CH_4_ reduction across primiparous and multiparous cows with 10 g/kg DM supplementation level (Wang et al., 2023). Methane emission can be influenced by diet composition, for example, forage:concentrate ratio and forage composition (Hristov et al., 2022). Therefore, differences in composition of the diets between studies might also explain differences in observed CH_4_ mitigation efficacy of nitrate supplementation. However, there is no evidence that nitrate was completely reduced to ammonia in the rumen, although a higher than 100% of CH_4_ mitigation potential was found in the current study. It is suggested that adding nitrate is toxic for rumen archaea as Asanuma et al. (2015) found a dramatic decline in the ruminal methanogen abundance in goats receiving nitrate. Therefore, the possible toxicity to methanogens of adding nitrate probably contributes to CH_4_ mitigation by reducing methanogen activity.

The increase in H_2_ indicated that not all the H_2_ redirected from methanogenesis was used in the nitrate reduction process. The increased H_2_ might also be ascribed to a reduced methanogen activity because of the toxic effect of nitrate on methanogens (Asanuma et al., 2015), thereby reducing the hydrogen consumption. In addition, the urea or nitrate supplementation might also induce diurnal variations in feeding behavior, especially right after feeding. The diurnal patterns of CH_4_ production (chambers) and CH_4_ volume proportion (rumen headspace) reflect each other (Figure 1b and 1c). This alignment probably implies that the methane emission from the hindgut is limited and not large enough to show a substantial impact on the methane emission from ruminal methane production. There were 4 time points where cows supplemented by urea showed higher hourly CH_4_ production from chamber measurement, but lower volume proportion of CH_4_ from rumen headspace measurement. This discrepancy within some time points might partly originate from pH fluctuations, as pH highly affects the dissolved CO_2_ in rumen fluid and thereby CO_2_ release to the headspace, which again affects the headspace composition (Maigaard et al., 2024). Maigaard et al. (2024) found that average headspace CH_4_ concentrations were highly correlated with CH_4_ yield (g/kg DMI) measured in respiration chambers. We believe headspace sampling combined with chamber measures can be a strong tool to get quantitative production data for minor gases and isotopes not measurable by the chamber instruments (such as N_2_O in the present study) or with concentrations below the detection limit in the chamber outflow. The fluctuating CO_2_ dissolving might mask the headspace composition for time points. However, CH_4_ concentrations in rumen fluid and headspace both correlated well with CH_4_ yield measured in chambers (Maigaard et al., 2024), and the headspace ratio to CH_4_ of a certain gas should be unaffected by CO_2_ fluctuations and is therefore assumed to be proper for quantification of daily production of such gases when CH_4_ is measured in chamber.

An interaction between nitrate supplementation and time on hourly CH_4_ production was noted, whereas neither time nor interaction between nitrate supplementation and time were significant in the rumen headspace data. Moreover, the increase in N_2_O production indicates that some of the ${NO}_{3}^{-}-N$ probably ended up in N_2_O, which is in agreement with Petersen et al. (2015). Although an increase in N_2_O in the rumen headspace was observed, a full picture of N_2_O emissions would require evaluating emissions from dung and urine, which were not measured in the current study.

Ruminal VFA proportions were unaffected in the current study, aside from a higher valerate proportion and a tendency for increased butyrate proportion for nitrate compared with urea supplementation. It has been reported that nitrate addition could promote the growth of some ruminal cellulolytic bacteria species, which also could improve valerate production in the rumen (Zhao et al., 2015). The effect of nitrate supplementation on VFA composition varies between different studies. Olijhoek et al. (2016) reported a tendency to a linear decrease in propionate proportion and a linear increase in butyrate when feeding 3 nitrate doses of 5.3, 13.6, and 21.1 g/kg DM. A higher acetate proportion and lower propionate proportion were observed in cows receiving nitrate addition levels of 22.5, 21.5, and 18 g/kg DM, respectively (Guyader et al., 2015; Troy et al., 2015). One reason for this variation is the sampling time. All studies that have shown a significant effect of nitrate addition on VFA had a sampling time within a few hours after feeding. However, Olijhoek et al. (2016) sampled at different time points evenly spread during the day and only found tendencies for effects. Li et al. (2012) did not find any effect of nitrate supplementation on rumen VFA when collecting rumen samples more than 6 h after feeding. The effects on nitrate supplementation on rumen VFA also followed the diurnal patterns of CH_4_ emission, rumen pH, and H_2_ pressure in the rumen caused by nitrate supplementation (Guyader et al., 2015; Olijhoek et al., 2016). In addition, the effect of nitrate supplementation also depends on the dose of supplementation as the CH_4_ mitigation of nitrate supplementation is a dose-dependent effect (Feng et al., 2020).

Rumen headspace gas data were corrected for varying ambient air contamination as the nitrogen concentration in our samples was considerably higher than the values (2%–4%) reported in a previous study (Moate et al., 1997). Therefore, the data in this study were calculated as volume proportion of the 3 gases in question instead of concentration. The reason for this contamination was probably that our sampling method did not completely prevent pollution with ambient air.

Use of nitrate as a CH_4_ mitigation tool induces a risk of increasing environmental and climate effects from the manure if nitrate is added as top dressing and not substituting other N sources in the diet as it then will increase N output especially in urine. However, further work is required to evaluate such trade-offs. Methane potential of the feces seems unaffected by nitrate addition (Maigaard et al., 2022).

In conclusion, with a dose of 8.6 g/kg DM, nitrate supplementation reduced CH_4_ production and yield with the largest effect on production just after feeding, and increased H_2_ production and yield compared with urea supplementation. However, nitrate supplementation also increased N_2_O production compared with urea supplementation.
